# Antiviral Activity of *Morus nigra* L. Molasses and an Anthocyanin‐Rich Extract Against Herpes Simplex Virus and Development of a Film Formulation

**DOI:** 10.1002/cbdv.71863

**Published:** 2026-11-09

**Authors:** Nuraniye Eruygur, Tuğsen Buyukyildirim, Sema Arisoy, Sercan Palanci

**Affiliations:** ^1^ Department of Pharmacognosy, Faculty of Pharmacy Selcuk University Konya Turkiye; ^2^ Department of Pharmaceutical Technology, Faculty of Pharmacy Selcuk University Konya Turkiye; ^3^ Kiraz Vocational School, Department of Laboratory and Veterinary Health Dokuz Eylul University Izmir Türkiye

**Keywords:** anthocyanins, antiviral activity, black mulberry, film formulation, Herpes Simplex Virus‐1 (HSV‐1), molasses, *Morus nigra* L

## Abstract

This study evaluated the in vitro antiviral efficacy of *Morus nigra* L. (black mulberry) molasses (BMM) and an anthocyanin‐rich extract (BME) incorporated into mucoadhesive films for potential topical treatment against herpes simplex virus (HSV). Recurrent orolabial herpes requires effective localized therapy. *Morus nigra* is rich in bioactive polyphenols, flavonoids, and anthocyanins known for potent antioxidant and antiviral properties. Mucoadhesive film delivery systems can enhance localized retention and patient compliance. BMM was obtained by thermal concentration of fresh fruit juice, while BME was produced via solid‐phase extraction and confirmed for high anthocyanin content using UV spectrophotometry. The antiviral activity of BMM or BME was assessed against HSV‐1‐infected Vero cell cultures. A mucoadhesive film formulation containing BMM or BME was developed by the solvent casting method. Both BMM and BME demonstrated significant antiviral activity. BMM at 0.225 mg/100 µL achieved approximately 81% inhibition against HSV‐1, while BME at 0.525 mg/100 µL achieved approximately 97.1% inhibition. The film formulations were successfully developed. Standardized *Morus nigra* molasses and anthocyanin‐rich extracts demonstrate potent in vitro HSV‐1 inhibition. Their successful incorporation into mucoadhesive films highlights their potential as innovative, nature‐based topical delivery systems for managing localized orolabial HSV infections.

## Graphical abstract

Quantitative standardization of *Morus nigra* L. fruit extract and molasses reveals significant total phenolic and anthocyanin contents. Polymer‐based film formulations incorporating these standardized preparations exhibit robust antioxidant capacity and pronounced antiviral efficacy against HSV‐1 and HSV‐2, offering a promising, biocompatible topical strategy to combat localized herpes simplex viral infections.

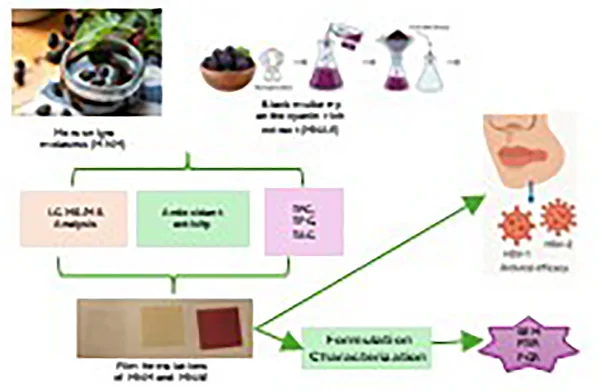

## 1 Introduction

Herpes simplex virus type 1 (HSV‐1) and HSV‐2 are highly prevalent human pathogens with worldwide prevalence levels of approximately 67% and 13%, respectively. The World Health Organization (WHO) estimates that more than four billion people are infected with HSV‐1 (3.7 billion cases) and HSV‐2 (400 million cases) [1, 2]. Both HSV‐1 and HSV‐2 are transmitted through close contact and cause lifelong infection. Most people acquire HSV‐1 early in life through the orolabial mucosa, while HSV‐2 infections are transmitted sexually [1]. Both HSV types persist lifelong by establishing latency within sensory neurons, especially in the trigeminal and sacral ganglia [3]. Antivirals—including acyclovir, valaciclovir, famciclovir, penciclovir, and cidofovir are the drugs used for the management of the illness. Especially acyclovir, a synthetic acyclic purine‐nucleoside analog, is the standard treatment for HSV infections [4]. Orolabial herpes is usually caused by HSV‐1, although a minority of cases are caused by HSV‐2. Topical formulations are mainly applied in the management of orolabial lesions and provide limited benefit, particularly when used at the early stages of reactivation. However, treatment choices for HSV infections are still limited, highlighting the need for alternative therapies. There is a significant need for the development of novel antiviral agents that operate through alternative mechanisms of action and incorporate effective application approaches [5].

*Morus nigra* L. (Moraceae) (black mulberry) is native to Southwest Asia. It has been cultivated throughout Europe and around the Mediterranean for centuries [6]. Black mulberry has demonstrated a wide range of pharmacological therapeutic effects, including antimicrobial, antimelanogenic, antidiabetic, anti‐obesity, antihyperlipidemic, antiviral, and anticancer activities [7]. When ethnopharmacological studies on black mulberry were examined, it was found that black mulberry fruits were used by the public for the treatment of oral diseases or aphthae in different regions of Türkiye. The topical application of black mulberry molasses has been documented as a traditional folk remedy for the treatment of herpes labialis lesions (cold sores) at the oral commissures in the pediatric population of Türkiye [8, 9, 10, 11, 12, 13, 14, 15]. In Pakistan, *M. nigra* is mixed with *Hippophae rhamnoides* L. and used as a syrup to treat bronchial congestion [16]. According to a previous study, an aqueous extract obtained from *Morus alba* leaves collected from a local garden in East Cairo, Egypt, between May and June 2014 exhibited marked antiviral activity against both HSV‐1 and HSV‐2 in infected Vero cells. Based on cytopathic effect (CPE) observations, the extract was active at 10 µg/mL, while CPE was detected at 3.5 µg/mL [5].

Creams or systemic drugs are frequently used in the current standard for the therapy of herpetic lesions in the oral cavity. Nevertheless, these formulations may encounter difficulties such as low localized drug concentration, short retention duration at the lesion site, and poor patient compliance. There is a strong need to create a novel, localized medication delivery mechanism to solve these problems and optimize the therapeutic advantages of black mulberry. Bioadhesive film formulations represent a superior strategy for topical delivery to herpetic lesions. These thin, flexible films offer several distinct advantages, such as prolonged contact time, localized, and sustained release, enhanced patient compliance, and protection of the lesion [5].

Previous studies investigating the effects of medicinal plants against HSV‐1 infection have used topical microemulsion [17] and nanoparticle formulations [18]. However, this study is the first to develop and evaluate the use of a mucoadhesive film formulation against HSV‐1 infection.

In recent decades, the rapid emergence of viral pathogens—ranging from pandemic influenza to novel coronaviruses—has highlighted the urgent need for broad‐spectrum antiviral compounds derived from natural sources [19, 20]. In this context, botanical extracts rich in bioactive phenolics and anthocyanins, such as black mulberry (*Morus nigra* L.), serve as promising candidates for novel natural antiviral and antioxidant formulations. The current study aims to evaluate the antiviral and antioxidant effects of an anthocyanin‐rich extract and molasses prepared from black mulberry fruits, considering an ethnopharmacological study, and to develop these into a topical mucoadhesive film formulation for easy application and enhanced treatment efficiency against oral‐labial HSV infections.

## 2 Material and Methods

### 2.1 Supply of Black Mulberry Molasses and Fruits

Black mulberry molasses (BMM) was obtained from a natural products market and used in the experiments. Fresh ripe fruits of black mulberry were collected from the Ilgin, Ihsaniye neighborhood (1100 m altitude) in the C4 Konya region, Türkiye, on July 15, 2024. Taxonomic identification and botanical authentication were performed by Prof. Dr. Osman Tugay. A voucher specimen has been deposited at the Herbarium of the Faculty of Pharmacy, Selçuk University for future reference and verification.

### 2.2 Preparation of Anthocyanin‐Rich Extract From Black Mulberry Fruits

Fresh black mulberry fruits were homogenized and extracted with 80% ethanol (pH = 6.0) for 24 h. After filtration, fresh solvent was added to the fruit residue, and the extraction process was repeated. The filtrates were combined and concentrated at 40°C using a rotary evaporator to obtain the Anthocyanin‐Rich Extract from Black Mulberry Fruits (BME) with a final extraction yield of 26% (w/w, relative to fresh fruit weight). Anthocyanin‐Rich Extract from Black Mulberry Fruits (BME) was obtained for further examinations.

### 2.3 Total Phenol Content (TPC) Analysis

TPC of BMM and BME was determined using the Folin–Ciocalteu method with some modifications [21]. A mixture of samples, deionized water, and Folin–Ciocalteu reagent was incubated for 3 min. Then, 20% sodium carbonate (Na_2_CO_3_) was added. After 30 min of incubation at room temperature, absorbance was measured at 765 nm using a UV spectrophotometer. Results were expressed as mg gallic acid equivalents (GAE) per gram fresh weight (fw) based on a gallic acid standard curve (y = 0.0011x + 0.1459, r^2^ = 0.9951).

### 2.4 Total Anthocyanin Content (TAC) Analysis

TAC of BMM and BME was analyzed using the pH differential method [22]. Samples were diluted with 500 µL of 0.4 M sodium acetate buffer (pH 4.5) and incubated at room temperature for 15 min. The absorbance of the low‐ and high‐pH samples was measured at 515 and 700 nm, respectively. Results were expressed as grams of cyanidin‐3‐glucoside equivalents per kilogram fresh weight. TAC was calculated as cyanidin‐3‐glucoside (CE) equivalent using the following equation:

$$\mathrm{TAC}\left(\mathrm{mg}\;\mathrm{Cyanidin}-3-\mathrm{glucoside}/L\right)=\left(A\times 445\times \mathrm{DF}\right)/26900$$

where 26900 Is the Molar Extinction Coefficient; DF; Dilution Factor; A Is the Measured Absorbance Difference;

$$A=\left(A_{515}-A_{700}\right)\mathrm{pH}1.0-\left(A_{515}-A_{700}\right)\mathrm{pH}4.5$$

### 2.5 Total Flavonoid Content (TFC) Analysis

TFC of BMM and BME was determined using the aluminum chloride colorimetric method [23]. 150 µL samples and 2% AlCl_3_ solution were mixed in a 96‐well plate and incubated at room temperature for 15 min. Absorbance was measured at 435 nm. Quercetin (QE) was used as the reference standard (y = 0.0077x + 0.1632, r^2^ = 0.992).

#### 2.5.1 2,2‐Diphenyl‐1‐picrylhydrazyl (DPPH) Radical Scavenging Activity Assay

The DPPH assay was performed spectrophotometrically using the methods of Clarke et al. [21]. BMM and BME samples were prepared at different concentrations. Each solution (2 mL) was mixed with 0.5 mL of 1 mM methanolic DPPH solution. After 15 min of incubation at room temperature, absorbance was measured against a blank at 517 nm. Butylated hydroxytoluene was used as a positive reference, while methanol served as a control.

#### 2.5.2 2,2′‐Azino‐bis (3‐ethylbenzothiazoline‐6‐sulfonic Acid) Diammonium Salt (ABTS) Radical Scavenging Activity Assay

The ABTS radical scavenging activity of BMM and BME was measured using the method described by Re et al. [24]. The ABTS•^+^ radical was generated by reacting 15 mL of 7 mM ABTS solution with 264 µL of 140 mM potassium persulfate, then incubating in the dark at room temperature for 16 h (stock solution). The working ABTS solution was prepared by diluting the stock with methanol to an absorbance of 0.70 ± 0.02 at 734 nm. In a 96‐well plate, 50 µL of different concentration samples were mixed with 100 µL of ABTS working solution. Absorbance was measured at 734 nm.

### 2.6 Ferric Reducing Antioxidant Power (FRAP) Assay

The FRAP assay is based on the reduction of ferric‐tripyridyl triazine (Fe(III)‐TPTZ) complex to the blue‐colored ferrous form (Fe(II) in the presence of antioxidants [23]. Fresh FRAP reagent was prepared by mixing 25 mL of 300 mM acetate buffer (pH 3.6), 2.5 mL of 10 mM TPTZ solution (in 40 mM HCl), and 2.5 mL of 20 mM FeCl_3_·6H_2_O solution. For the test, 1 mL of the BMM and BME samples was mixed with freshly prepared FRAP reagent. The absorbance of the blue complex, indicating the sample's reducing power, was measured at 593 nm. Results were expressed as Trolox equivalents (TE, 6‐hydroxy‐2,5,7,8‐tetramethylchroman‐2‐carboxylic acid) in µmol TE/g fresh weight.

### 2.7 LC‐MS/MS Analysis

A total of fifty‐six phytochemicals were quantitatively analyzed using an LC–MS/MS system, consisting of a Shimadzu Nexera ultra‐high‐performance liquid chromatography (UHPLC) unit coupled with a tandem mass spectrometer. The chromatographic conditions applied in this study were based on previously reported methods [25, 26].

### 2.8 Film Formulation With BMM and BME

#### 2.8.1 Preparation of Formulation

Film formulations containing BMM and BME were prepared using the solvent‐casting technique. Briefly, 6.4 mL of a 5% (w/v) hydroxypropyl methylcellulose (HPMC) solution was transferred into a beaker, followed by the gradual addition (dropwise) of 0.5 mL of BMM, BME or distilled water, as detailed in Table 1. Subsequently, 0.145 mL of propylene glycol was incorporated as a plasticizer, and 3 mL of a 1% (w/v) chitosan solution was added. The resulting dispersions were magnetically stirred at 200 rpm until a uniform gel was obtained. To eliminate entrapped air bubbles, each formulation was sonicated in an ultrasonic bath at room temperature for 30 s. The degassed gels were then poured into 10 cm diameter Petri dishes and dried at 37°C for 24 h. The dried films were carefully peeled off and cut into 1 cm × 1 cm specimens for subsequent analyses.

**TABLE 1:** Formulation variables.

Formulation	Variable
**F1**	BMM
**F2**	BME
**F3**	Distilled water

#### 2.8.2 Characterization of Formulation

##### 2.8.2.1 Weight Variation

The film specimens (1 cm × 1 cm) from each formulation were weighed individually, and the mean weight and standard deviation were calculated to assess uniformity [27].

##### 2.8.2.2 Dissolution Time

Three film samples from each formulation were immersed in phosphate‐buffered saline (PBS, pH 7.4) at 37°C, and their complete dissolution times were recorded [27].

##### 2.8.2.3 pH Measurement

Film specimens were soaked in 20 mL of distilled water, and the surface pH of each dispersion was determined using a pH meter [27].

##### 2.8.2.4 Appearance

Macroscopic features, including color, transparency, and surface uniformity, were visually assessed for all film formulations [27].

##### 2.8.2.5 In Vitro Release Study

Film samples of known weight were immersed in 10 mL of distilled water and maintained at 37°C under constant stirring (50 rpm). Aliquots of 1 mL were withdrawn at predetermined intervals (5, 15, 30 min, and 1 h), replaced with fresh medium, and analyzed for anthocyanin content as a function of time [27].

##### 2.8.2.6 Thickness and Morphology

Film thickness and surface morphology were examined using Scanning Electron Microscopy (SEM). Representative images of F1, F2, and F3 films were captured to evaluate surface characteristics and cross‐sectional structure. The mean film thickness and standard deviation were recorded [27].

##### 2.8.2.7 FTIR Analysis

Fourier Transform Infrared (FTIR) spectra of the films were obtained using an ATR‐FTIR spectrometer (Perkin Elmer Spectrum 100, ZnSe crystal) in the 4000–400 cm^−1^ wavenumber range to identify potential interactions among formulation components [28].

#### 2.8.3 In Vitro Antiviral Activity Against HSV‐1

##### 2.8.3.1 Cell Culture

Vero (CCL‐81) cells were amplified in F12 Ham/Dulbecco's Modified Eagle Medium (DMEM) at 37°C in an incubator containing 5% CO_2_. The medium was supplemented with 10% fetal bovine serum (FBS), 1% antibiotic, and 1% L‐glutamine. During antiviral assays, the concentration of FBS in the culture medium was reduced to 2%.

##### 2.8.3.2 Concentration–Dose Studies

The cytotoxicity of the tested samples was evaluated in Vero cell cultures using a CCK‐8 cell viability assay, and the half‐maximal cytotoxic concentration (CC_50_) values were determined accordingly. All procedures were conducted in accordance with the manufacturer's protocol. Briefly, 100 µL of cell suspension, adjusted to the recommended cell density, was seeded into each well of 96‐well plates and incubated overnight at 37°C in a humidified atmosphere containing 5% CO_2_. After incubation, the culture medium was carefully removed and replaced with 100 µL of sample solutions prepared at different concentrations (10, 5, 2.5, and 1.25 mg per 100 µL) in medium supplemented with 3% FBS. Each concentration was tested in triplicate. Control wells containing only cell culture medium without test samples were included as cell controls, while additional blank wells were prepared to account for background absorbance. For background correction, wells without cells containing either medium alone or sample dilutions were included. Following treatment, the plates were incubated for 48 h under standard culture conditions. Subsequently, 10 µL of CCK‐8 reagent was added to each well, and the plates were further incubated for 2 h at 37°C in a 5% CO_2_ incubator. Optical density (OD) values, corresponding to absorbance, were measured at 450 nm using a microplate reader. Each experiment was performed in triplicate and repeated independently three times. The calculation of cytotoxicity (CC) was carried out using the formula provided below.

$$\begin{array}{ccc} & & \mathrm{Cc}\left(\%\right)=[\left(Od_{\mathrm{control}}-Od_{\mathrm{control}\mathrm{blank})}-\left(Od_{\mathrm{sample}}-Od_{\mathrm{sample}\mathrm{blank}}\right)\right]\\ & & /\left(Od_{\mathrm{control}}-Od_{\mathrm{control}\mathrm{blank}}\right)\times 100 \end{array}$$

Cytotoxicity assays were performed in triplicate, and results were expressed as mean ± standard deviation (SD). Graphs were constructed using Microsoft Excel by plotting cytotoxicity (%) against concentration, with error bars representing standard deviation.

The half‐maximal cytotoxic concentration (CC_50_) was defined as the concentration causing a 50% reduction in cell viability. CC_50_ values were estimated from the concentration–response data using regression modeling with CurveExpert Professional software. The Morgan–Mercer–Flodin (MMF) model provided the best fit for the P formulation, whereas the DR‐Hill‐Zerobackground model was selected for the black mulberry extract (E) based on goodness‐of‐fit criteria.

##### 2.8.3.3 Preparation of Penciclovir as a Positive Control and Determination of the Working Concentration

Penciclovir was used as a positive control to confirm the susceptibility of the HSV‐1 KOS strain used in this study to a reference antiviral compound and to verify that the antiviral assay system performed as expected. Penciclovir in crystalline powder form was dissolved in dimethyl sulfoxide (DMSO) to prepare a 100 mM stock solution. The stock solution was aliquoted into single‐use 100 µL volumes and stored at −20°C until use.

To determine an appropriate working concentration of penciclovir for use in Vero cells, penciclovir solutions at concentrations of 2.2, 1.8, 1.4, and 1.0 mM were prepared by diluting the stock solution in cell culture medium. The cytotoxic effects of penciclovir at these concentrations were evaluated using the CCK‐8 cell viability assay as described in the “Concentration–Dose Studies” section. Each concentration was tested in four replicates.

##### 2.8.3.4 Virus Titration Assay

To determine the infectivity of HSV‐1 used in the study, the microtitration method described in the literature was applied [29]. For this purpose, the assay was conducted in 96‐well plates. Tenfold serial dilutions of HSV‐1 were prepared in a logarithmic manner. Briefly, 100 µL of medium was added to all wells except the control groups. Subsequently, 11 µL of virus suspension was added to the initial wells designated for dilution. Serial dilutions were then performed by transferring 11 µL from each well to the next, using a fresh pipette tip at each step. Following the dilution process, 50 µL of cell suspension containing 40 000 cells per well in 10% FBS‐supplemented medium was added to all wells. The plates were incubated at 37°C in a humidified atmosphere with 5% CO_2_. During the 72‐h incubation period, the cells were monitored daily for the development of cytopathic effects (CPE). At the end of the incubation period, the results were evaluated, and the viral infectivity titer was calculated using a standard method reported in the literature [29].

##### 2.8.3.5 Verification of HSV‐1 in Vero Cells

In this study, HSV‐1 (KOS strain) amplification in Vero cells was confirmed by conventional PCR through the detection of viral nucleic acids in the culture medium. PCR amplification was carried out using primer pairs targeting the UL‐30 gene region, which has been reported to play a key role in HSV‐1 replication. For the PCR analyses, the Solis Biodyne FIREPol Master Mix with 12.5 mM MgCl_2_, 5x reaction kit was used. The thermal cycling conditions were determined based on the optimal values recommended by the manufacturer. The oligonucleotide sequences of the primer sets used in this study are presented in Table 2, while the PCR cycling conditions and reaction mixtures are provided in Table 3.

**TABLE 2:** Primer sequences used in the study.

Gene region	Primer sequence	Amplicon (bp)
**UL30**	**F**: 5’‐GAGGACGAGCTGGCCTTTCCG‐3’	**1810**
	**R**: 5’‐AAAACAGCAGGTCGACCAGGGC‐3’	

**TABLE 3:** Reaction mixtures and temperature cycles.

Mix	Amount (µl) Total 40 µl	PCR cycle		
		Celsius (°C)	Time	Cycle
5x Master Mix	8	95 (Initial denaturation)	7 min	1
Forward primer10pmol	1	95 (Denaturation)	30 s	30
Revers primer 10pmol	1	58 (Annealing)	60 s	
Distilled water	26	72 (Extension	2 min	
DNA	4	72 (Final extension)	7 min	1

At the end of the reaction, the PCR products were loaded onto a pre‐prepared 2% agarose gel and subjected to electrophoresis at 100 V and 80 mA for 50 min. Following electrophoresis, the products were visualized and evaluated using a gel imaging system.

##### 2.8.3.6 Determination of Antiviral Activity

Vero cells were seeded into 25 cm^2^ flasks and incubated overnight at 37°C in a humidified 5% CO_2_ atmosphere. Flasks reaching ≥80% confluency were selected for the experiments. The growth medium was removed, and cells were pretreated with test samples prepared in medium containing 5% FBS at concentrations below their CC_50_ values. These concentrations below the respective CC_50_ values were selected to minimize the potential influence of sample‐related cytotoxicity on viral titer results. BME and BMM were applied at 0.525 mg/100 µL and 0.225 mg/100 µL, respectively. A blank formulation (F3) was included at an equivalent concentration (0.375 mg/100 µL) to evaluate any intrinsic effect of the formulation matrix. Cells were incubated with these samples at 37°C in 5% CO_2_ to allow cellular interaction before viral exposure.

Following the pretreatment period, the sample‐containing media were removed, and the cells were infected with HSV‐1 (100 TCID_50_; 10^−^^4^ dilution) using a standard adsorption procedure. Following virus adsorption, fresh medium containing 5% FBS and the corresponding formulation was added to the BME‐, BMM‐, and F3‐treated groups, and incubation was continued. The virus control group received fresh medium containing 5% FBS only. The cell control group received neither virus nor any formulation.

Cells were then incubated for 72 h at 37°C in a 5% CO_2_ atmosphere and monitored daily under an inverted microscope for cytopathic effects (CPE). The experiment was terminated on day 3. All assays were performed in four independent replicates. Subsequently, virus titration assays were conducted to determine changes in HSV‐1 infectivity, and results were compared with untreated virus controls to evaluate the antiviral activity of the tested samples [29].

Antiviral activity was evaluated by determining the viral inhibition rate based on TCID_50_ values. All experiments were performed in four independent replicates at different time intervals, and the results were expressed as arithmetic mean values. The viral inhibition rate (%) was calculated using the following equation:

$$\mathrm{Viral}\mathrm{Inhibition}\left(\%\right)=\left[\left(V_{0}-\mathrm{Vt}\right)/V_{0}\right]\times 100$$

where V_0_ represents the TCID_50_ value of untreated virus (control), and Vt represents the TCID_50_ value of virus treated with the test compounds.

### 2.9 Statistical Analysis

To evaluate the statistical significance of antiviral activity among BMM, BME, and F3, a one‐way Analysis of Variance (ANOVA) followed by Tukey's post‐hoc test was performed. Experimental data were collected in triplicate (n=3) and expressed as mean ± standard deviation (SD). Statistical significance was defined as p<0.05. All statistical analyses were conducted using IBM SPSS Statistics (version 30.0).

## 3 Results

### 3.1 Antioxidant Activity

Phytochemical contents and antioxidant activities of BMM and BME are given in Table 4. BME was quantitatively standardized based on its main bioactive constituent classes: Total Phenolic Content (TPC) at 4.49 ± 1.24 mg GAE/g extract, Total Flavonoid Content (TFC) at 0.40 ± 0.20 mg QE/g extract, and Total Anthocyanin Content (TAC) at 0.595 ± 0.047 mg CE/g extract. BMM was characterized by its Total Anthocyanin Content (TAC) of 0.020 ± 0.013 mg CE/g. In the DPPH and ABTS radical scavenging activity determinations, black mulberry fruit (DPPH: 35.53%, ABTS: 70.49%) was found to have higher antioxidant activity than molasses (DPPH: 17.5%, ABTS: 9.05%). In the FRAP method, black mulberry molasses (0.0147 µg/g Trolox equivalent) showed higher activity than the fruit extract (0.0047 µg/g Trolox equivalent).

**TABLE 4:** TPC, TFC, and TAC amounts, and antioxidant activities of Black mulberry samples.

Samples	TPC (mg GAEs/g)^a^	TFC (mg QEs/g)^b^	TAC (mg CEs/g)^c^	FRAP (mg TE/g)^d^	Radical scavenging activity (inhibition%)^e^	
					DPPH	ABTS
BMM	—^f^	—	0.020 ± 0.013	0.0147	17.57 ± 1.97	9.05 ± 1.69
BME	4.49 ± 1.24	0.40 ± 0.20	0.595 ± 0.047	0.0047	35.53 ± 3.42	70.49 ± 4.64 (IC_50_ = 225.50 ± 8.50 µg/mL)
BHT	—	—	—	—	65.34 ± 2.28	—
Trolox	—	—	—	—	–—	86.20 ± 2.61

^a^GAEs. Gallic acid equivalents.

^b^QEs. Quercetin equivalents.

^c^Cyanidin‐3‐glucoside equivalents.

^d^TE: Trolox equivalents.

^e^1 mg/mL stock concentration.

^f^No result.

Pearson correlation analysis was performed to assess potential linear relationships among TPC, TFC, TAC, DPPH, and antiviral activity. A moderate positive correlation was observed between TPC and antiviral activity (r=0.515), whereas TFC, TAC, and DPPH showed moderate inverse correlations (r=−0.515). However, due to the limited sample size (n=3), these linear correlations did not reach statistical significance (p>0.05). Further study with an expanded sample set is required to establish conclusive dose‐dependent relationships.

### 3.2 LC‐MS/MS Analysis Results

In the LC‐MS/MS analysis, qualitative identification and quantification of secondary metabolites in Black Mulberry Extract (BME) were carried out according to the analytical strategy described by Yilmaz et al. [25]. Secondary metabolites were assigned based on a multi‐tiered diagnostic identification protocol. Key phenolic compounds were unambiguously identified by comparing their retention times (t_R_), precursor ion *m*/*z* values, and collision‐induced dissociation (CID) tandem MS/MS product spectra directly with authentic commercial reference standards analyzed under identical analytical conditions. 53 phenolic compounds were analyzed in BMM and BME. The analytical method validation parameters of the LC‐MS/MS method and phytochemical composition of samples are given in Table 5. According to the LC‐MS/MS analysis results, there are significant differences between the phenolic compound profiles of BMM and BME. Quinic acid (9.936 µg/g) and a low amount of hesperidin (0.023 µg/g) were detected in BMM. In contrast, BME exhibited a richer phenolic compound content. Especially quinic acid (252.086 µg/g) was found in high amounts, while flavonoids and phenolic compounds such as chlorogenic acid, (1.415 µg/g) protocatechuic acid, (0.804 µg/g) caffeic acid, (0.029 µg/g) rutin, (0.081 µg/g) isoquercitrin, (0.054 µg/g) quercetin, (0.169 µg/g) hesperidin, (0.327 µg/g) naringenin, (0.006 µg/g) hesperidin (0.016 µg/g) and kaempferol (0.014 µg/g) were also detected. LC‐MS/MS chromatograms are shown in Figures S1–S3.

**TABLE 5:** LC‐MS/MS analysis results of BMM and BME.

No.	Analits	Rt^a^	M.I. (*m*/*z*)^b^	F.I. (*m*/*z*)^c^	İon mode	Equation	*r^2^* ^d^	*RSD*%^e^		Linearity range (mg/L)	LOD/LOQ (µg/L)^f^	Recovery (%)		*U* ^g^	mg analyte/g extract	
								Interday	Intraday			Interday	Intraday		Black mulberry molasses	Black mulberry extract
1	Quinic acid	3.0	190.8	93.0	Negative	*y* = −0.0129989+2.97989*x*	0.996	0.69	0.51	0.1–5	25.7/33.3	1.0011	1.0083	0.0372	9.936	252.086
6	Protocatechuic acid	6.8	152.8	108.0	Negative	*y* = 0.211373+12.8622*x*	0.957	1.43	0.76	0.1–5	21.9/38.6	0.9972	1.0055	0.0350	N.D.	0.804
9	Chlorogenic acid	8.4	353.0	85.0	Negative	*y* = 0.289983+36.3926*x*	0.995	2.15	1.52	0.1–5	13.1/17.6	1.0000	1.0023	0.0213	N.D.	1.415
17	Caffeic acid	12.1	179.0	134.0	Negative	*y* = 0.120319+95.4610*x*	0.999	1.11	1.25	0.05–2.5	7.7/9.5	1.0015	1.0042	0.0152	N.D.	0.029
33	Rutin	25.6	608.9	301.0	Negative	*y* = −0.0771907+2.89868*x*	0.999	1.38	1.09	0.1–5	15.7/22.7	0.9977	1.0033	0.0247	N.D.	0.081
34	Isoquercitrin	25.6	463.0	271.0	Negative	*y* = −0.111120+4.10546*x*	0.998	2.13	0.78	0.1–5	8.7/13.5	1.0057	0.9963	0.0220	N.D.	0.054
35	Hesperidin	25.8	611.2	449.0	Positive	*y* = 0.139055+13.2785*x*	0.999	1.84	1.35	0.1–5	19.0/26.0	0.9967	1.0043	0.0335	0.023	0.327
47	Quercetin	35.7	301.0	272.9	Negative	*y* = +0.00597342+3.39417*x*	0.999	1.89	1.38	0.1–5	15.5/19.0	0.9967	0.9971	0.0175	N.D.	0.169
48	Naringenin	35.9	270.9	119.0	Negative	*Y* = −0.00393403+14.6424*x*	0.999	2.34	1.69	0.1–5	2.6/3.9	1.0062	1.0020	0.0392	N.D.	0.006
49	Hesperetin	36.7	301.0	136.0/286.0	Negative	*y* = +0.0442350+6.07160*x*	0.999	2.47	2.13	0.1–5	7.1/9.1	0.9998	0.9963	0.0321	N.D.	0.016
52	Kaempferol	37.9	285.0	239.0	Negative	*y* = −0.00459557+3.13754*x*	0.999	1.49	1.26	0.05–2.5	10.2/15.4	0.9992	0.9990	0.0212	N.D.	0.014

abbreviation: N.D., not detected.

^a^R.T.: Retention time.

^b^MI (*m*/*z*): Molecular ions of the standard analytes (m/z ratio).

^c^FI (*m*/*z*): Fragment ions.

^d^ *r* ^2^: Coefficient of determination.

^e^RSD: Relative standard deviation.

^f^LOD/LOQ (µg/L): Limit of detection/quantification.

^g^U (%): percent relative uncertainty at 95% confidence level (*k* = 2).

### 3.3 Formulation Characterization

Comprehensive characterization of the film formulations (F1–F3) was performed to elucidate how the incorporation of BMM and BME influences the physicochemical integrity, surface morphology, and release behavior of HPMC–chitosan‐based films. These evaluations aimed to determine the suitability of the developed films as natural, bioactive‐containing polymeric systems for potential orolabial application of BMM and BME. The results are presented in Table 6, Figures 1, and 2 below. SEM images are presented in Figure 3, and FTIR results are presented in Figure 4.

**TABLE 6:** Characterization of formulations.

Formulation	Weight variation (mg)	Disintegration time (s)	pH
**F1**	8.27 ± 1.01	56 ± 3	5.36 ± 0.01
**F2**	7.73 ± 1.35	101 ± 12	5.14 ± 0.02
**F3**	4.43 ± 1.40	22 ± 5	5.71 ± 0.01

**FIGURE 1:**
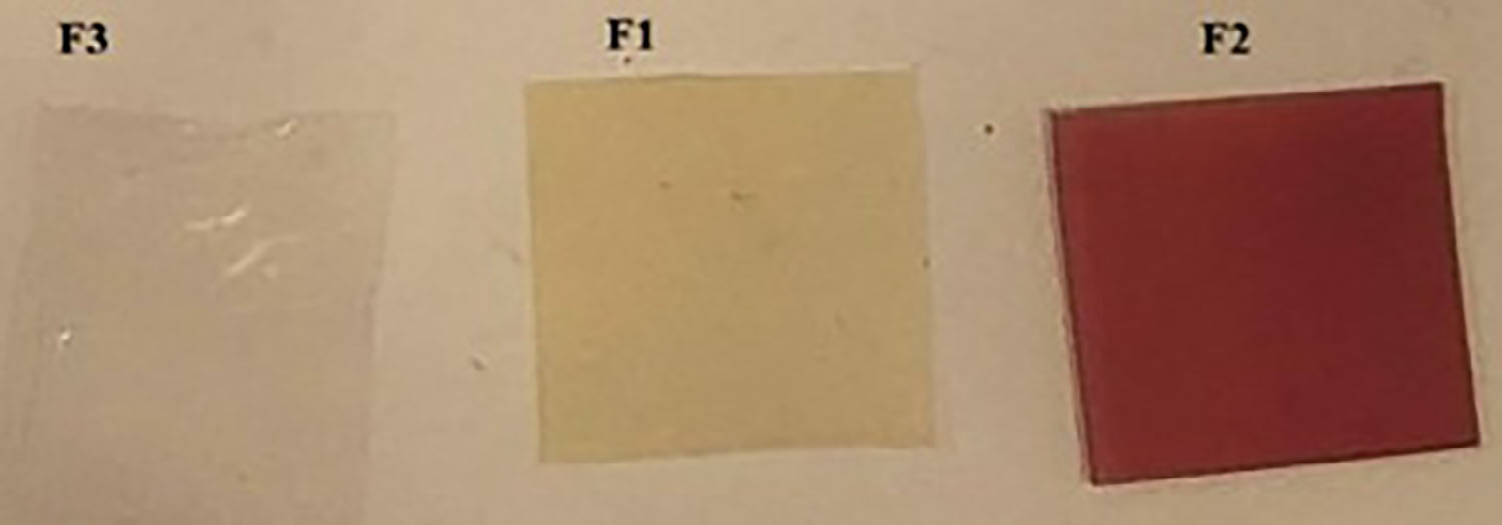
Images of film formulations.

**FIGURE 2:**
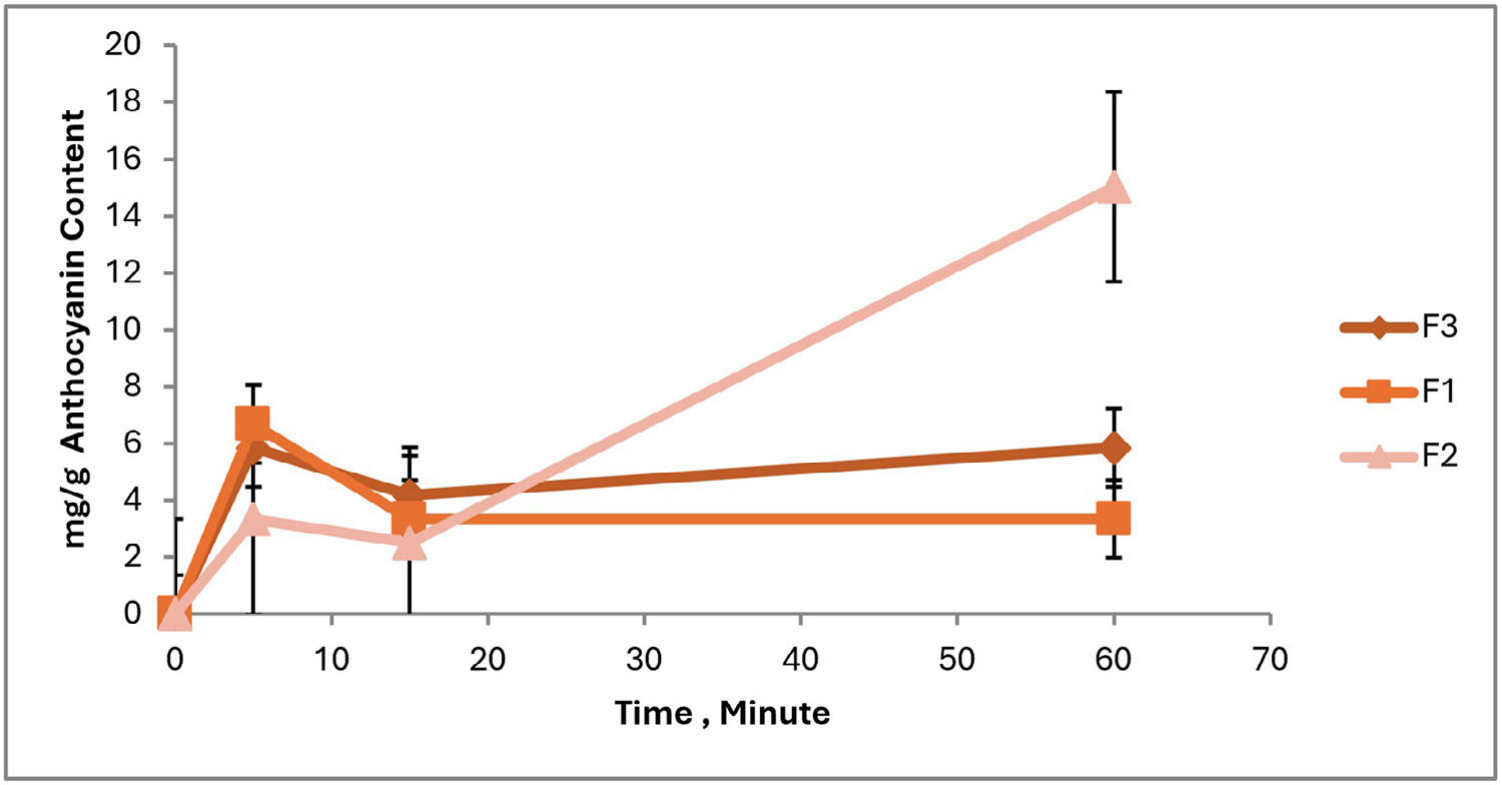
Anthocyanin content released over time.

**FIGURE 3:**
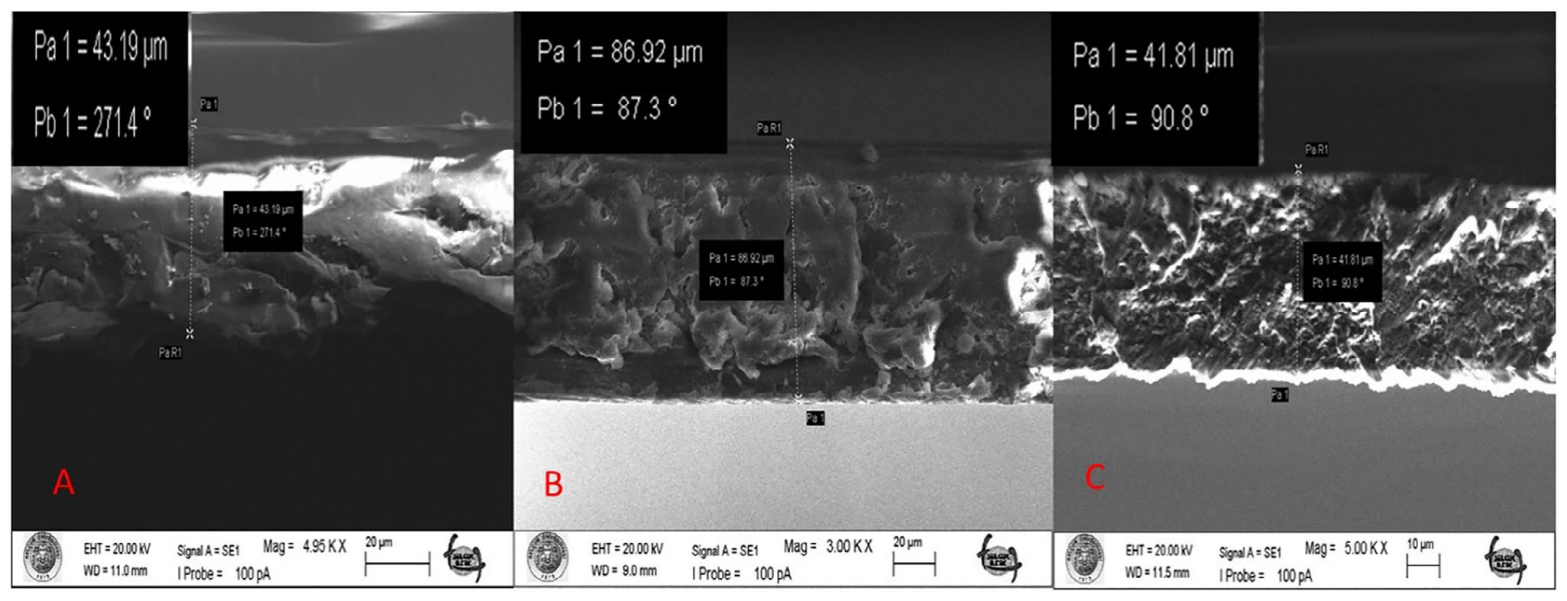
SEM images of formulations.

**FIGURE 4:**
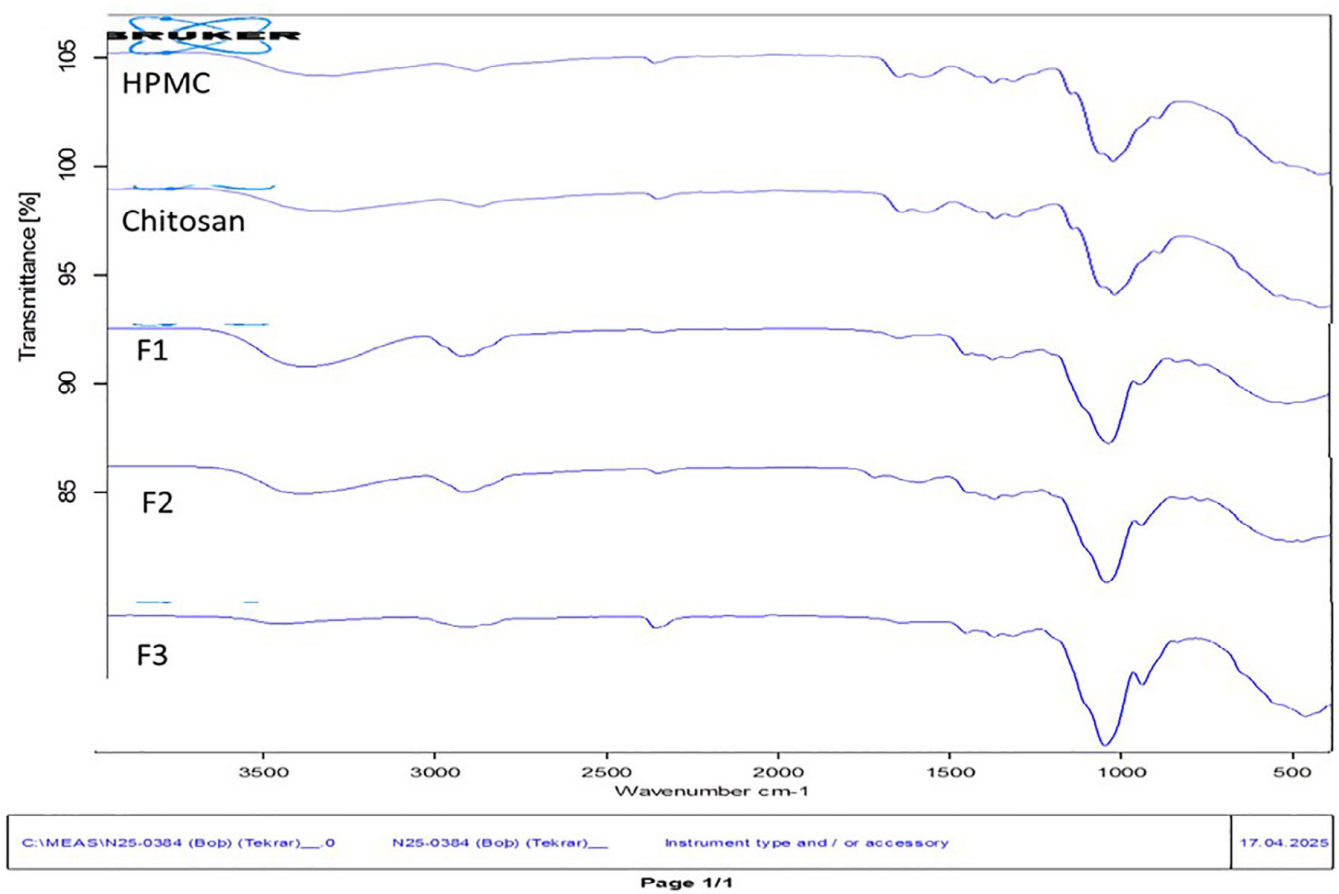
FTIR spectrum of F1, F2, F3, chitosan and HPMC.

### 3.4 Results of In Vitro Antiviral Activity Against Herpes Virus

#### 3.4.1 Cytotoxicity Analyses

The cytotoxic effects of BMM and BME on Vero cells are presented in Figure 5. Results were expressed as mean ± SD (*n* = 3).

**FIGURE 5:**
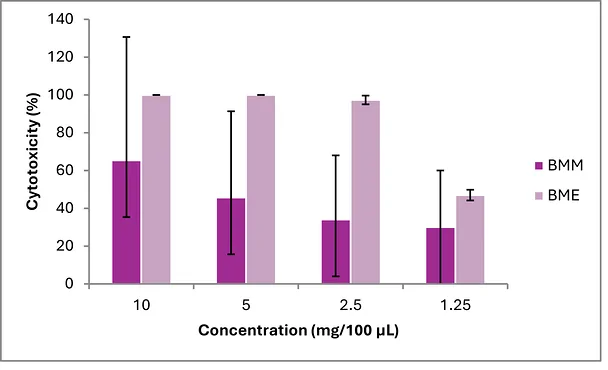
Cytotoxicity of Black Mulberry Molasses (BMM) and Anthocyanin‐Rich Extract (BME) on Vero cells.

The regression analysis of black mulberry molasses demonstrated a strong linear correlation (*r* = 1.0000), with a calculated CC_50_ value of 5.83 mg/100 µL. Similarly, the anthocyanin‐rich extract exhibited a high degree of correlation (*r* = 0.9999), with a lower CC_50_ value of 1.26 mg/100 µL, indicating higher cytotoxic potency compared to the molasses. Based on the cytotoxicity findings, concentrations below the respective CC_50_ values were selected for the antiviral assays to reduce the potential contribution of sample‐related cytotoxicity.

#### 3.4.2 Verification of HSV‐1 in Infected Vero Cells

HSV‐1 amplification in Vero cells was confirmed by conventional PCR targeting the UL30 gene region. As shown in Figure 6, a distinct amplicon of approximately 1810 bp was detected in the virus‐containing samples, consistent with the expected product size. No amplification was observed in the negative control, confirming the specificity of the assay.

**FIGURE 6:**
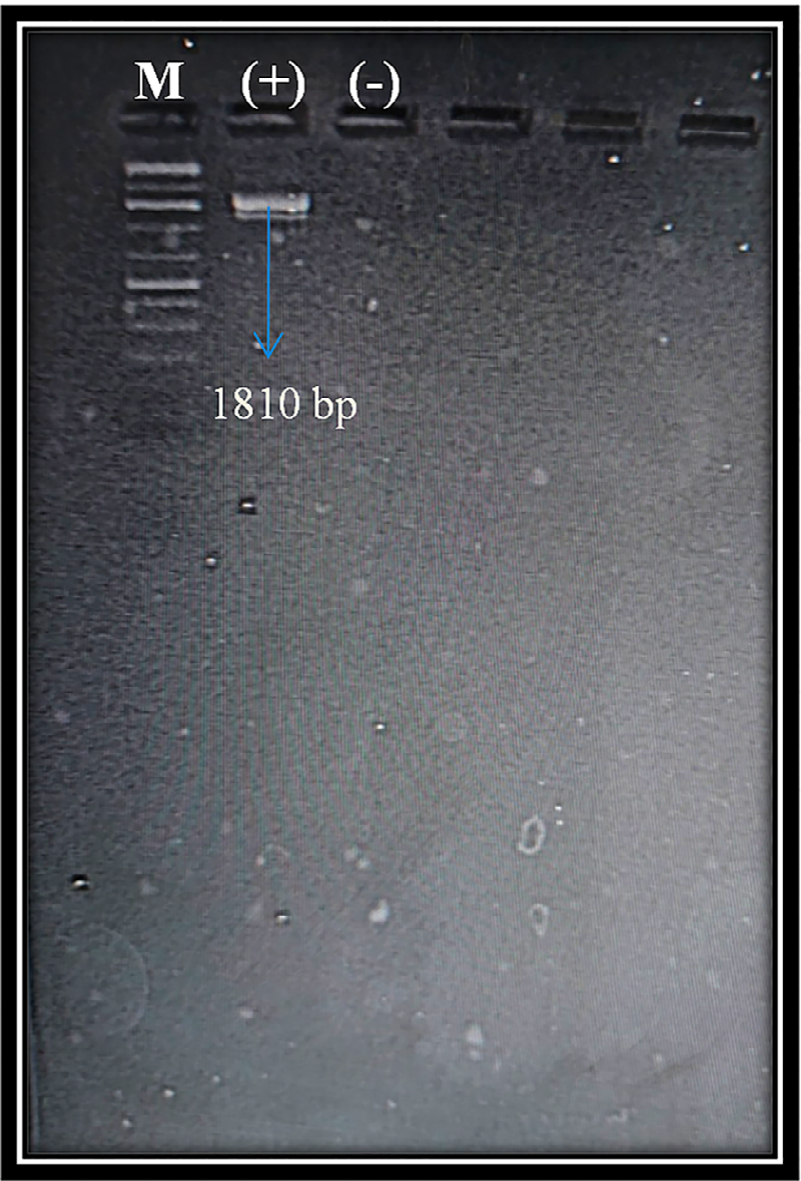
Agarose gel electrophoresis of PCR products targeting the HSV‐1 UL30 gene region. M: 1 kb DNA ladder; (+): positive control; (−): negative control.

#### 3.4.3 Antiviral Activity Test Results

Based on the calculations, it was determined that the BMM at a concentration of 0.225 mg/100 µL exerted approximately 81% viral inhibition, whereas the BME at a concentration of 0.525 mg/100 µL demonstrated an antiviral effect of approximately 97.1% against HSV‐1 (Table 7). Examination of the control groups revealed that the unloaded formulation (F3), at a concentration of 0.375 mg/0.1 mL, did not exhibit any antiviral or cytotoxic effect. Statistical analysis showed significant values of *p* = 0.0042 for the BMM and *p* = 0.0010 for the BME.

**TABLE 7:** Changes in HSV‐1 viral titers in Vero cells following treatment with Black Mulberry Molasses (BMM) and Anthocyanin‐Rich Extract (BME). Viral titers were expressed as TCID_50_/mL, and the data are presented as the arithmetic mean ± standard deviation (SD) of four replicates (n = 4).

Group	1. Trial	2. Trial	3. Trial	4. Trial	Arithmetic mean ± SD
**BMM**	6.67 × 10^6^	1.19 × 10^7^	2.11 × 10^6^	2.11 × 10^6^	**(5.70** ± **4.66)** × **10^6^**
**BME**	6.67 × 10^5^	2.11 × 10^6^	2.11 × 10^5^	3.75 × 10^5^	**(8.41** ± **8.67)** × **10^5^**
**HSV‐1 (Vc)**	3.75 × 10^7^	2.11 × 10^7^	3.75 × 10^7^	2.11 × 10^7^	**(293** ± **0.95)** × **10^7^**

Abbreviation: Vc, virus control.

Based on the calculated statistical significance values, we conclude that the compounds used exhibit significant antiviral activity. Microscopic images of antiviral activity studies are shown in Figure 7, syncytial cell forms are clearly seen in Figures C and E. Figure D: CPE‐induced plaque images formed by HSV‐1 are shown in the circle. Graphical representations of the microtitration test results are shown in Figures 8 and 9.

**FIGURE 7:**
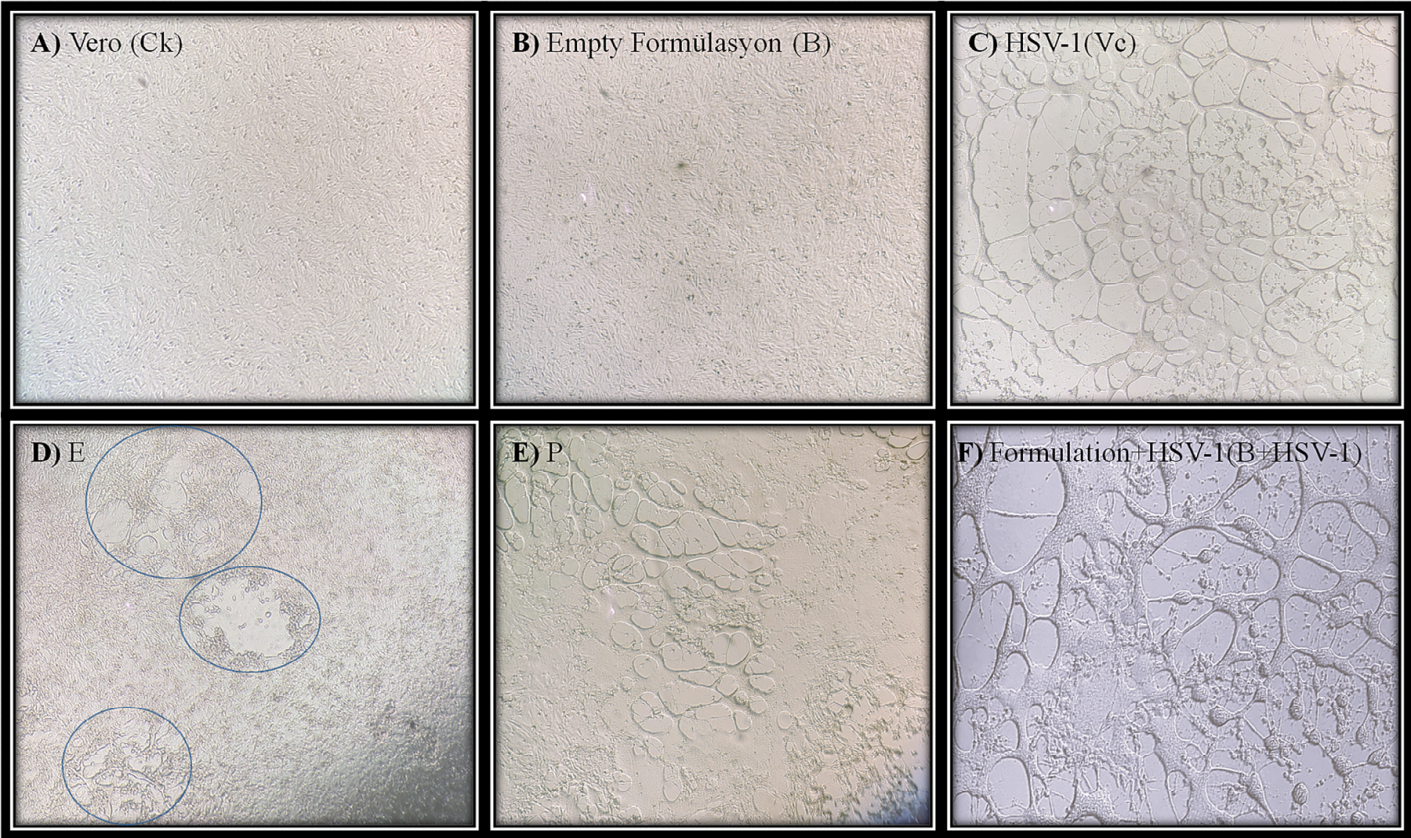
Microscopic images of the antiviral efficacy studies at 48 h (x60 magnification). (A) Vero cell control. (B) Empty formulation used in the study, not loaded with the active substance(F3). (C) HSV‐1 control group not under substance pressure. (D) BME(E); (E) BMM (P); (F) CPE images resulting from HSV‐1 infection of cells treated with the empty formulation.

**FIGURE 8:**
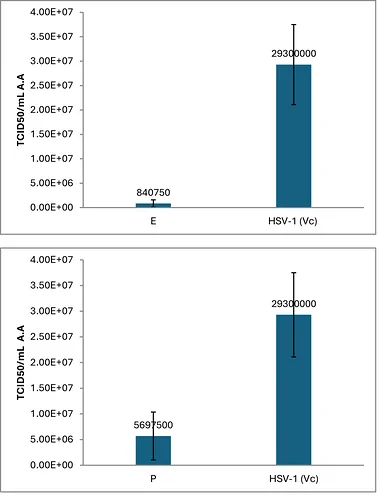
Viral load values of HSV‐1 treated and untreated with the substance (Virus control: Vc) in TCID50/mL. E: BME. P: BMM. ±: Standard deviation (SD). A.A: Arithmetic means of viral titers obtained from 4 replicates.

**FIGURE 9:**
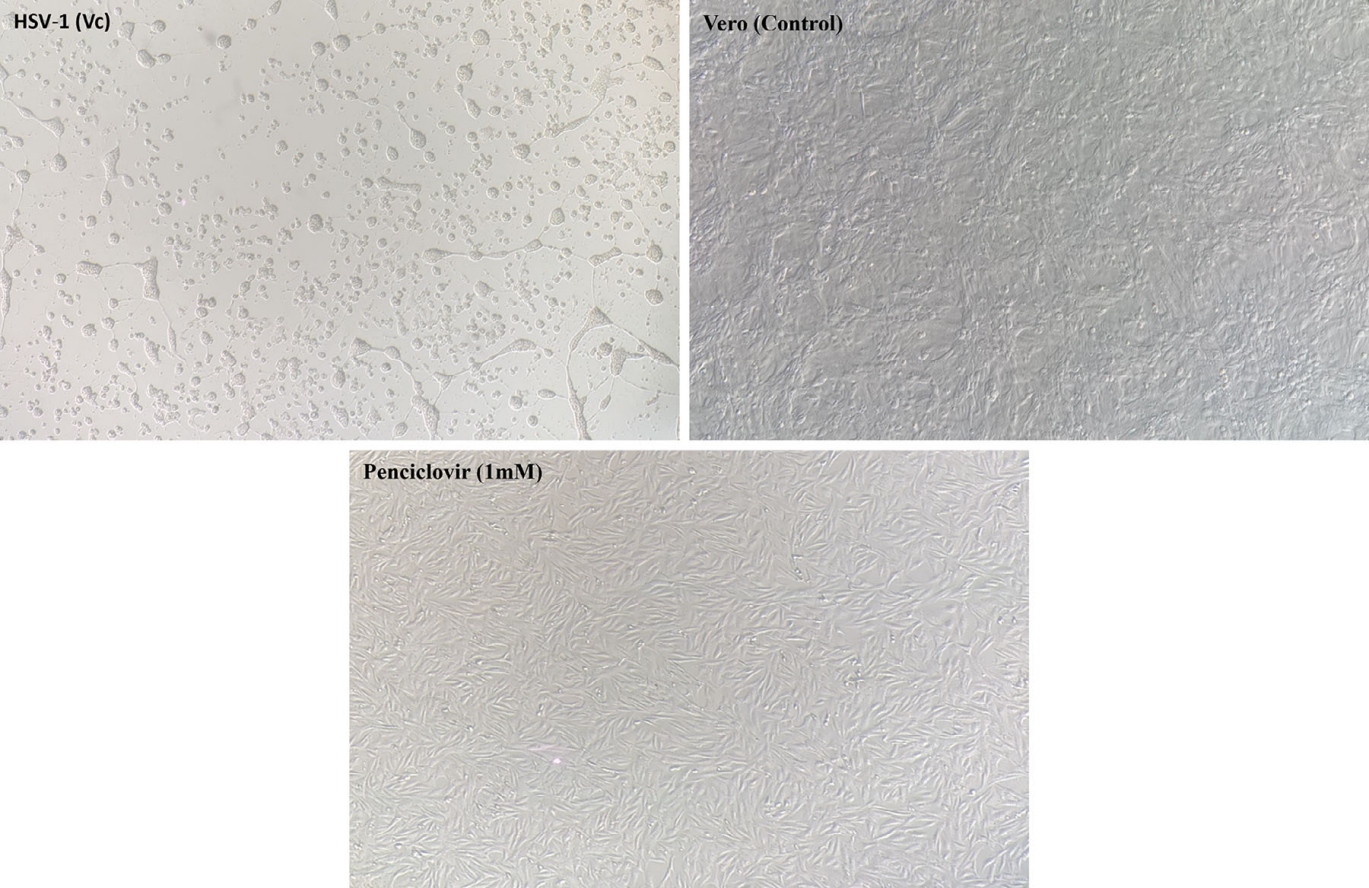
Representative microscopic images obtained at 72 h of the experiment. VC: virus control (×60).

#### 3.4.4 Cytotoxicity and Antiviral Activity Results of the Penciclovir Positive Control

The mean percentages of viable cells following treatment with penciclovir at concentrations of 2.2, 1.8, 1.4, and 1.0 mM were 53.36 ± 5.74%, 92.46 ± 17.77%, 99.57 ± 9.71%, and 112.59 ± 10.42%, respectively. Viability values exceeding 100% indicate that the CCK‐8 absorbance in the corresponding wells exceeded the mean of the untreated cell control and were therefore interpreted as evidence of the absence of marked cytotoxicity.

Penciclovir was tested after dilution of the 100 mM stock solution prepared in DMSO into the cell culture medium. Consequently, each penciclovir evaluation also contained the corresponding final concentration of DMSO derived from the stock solution. Therefore, the purpose of this assay was not to separately determine the cellular effects of penciclovir and DMSO, but rather to evaluate the overall cytotoxic effect of the final penciclovir working solution to be used in the antiviral experiments on Vero cells.

Based on these findings, 1.0 mM penciclovir, which exhibited low cytotoxicity, was selected as the positive control concentration for the antiviral experiments. Accordingly, a separate assessment of DMSO‐specific cytotoxicity was not considered necessary.

The antiviral activity of penciclovir at a concentration of 1.0 mM was evaluated as described previously in the “Determination of Antiviral Activity” section. In experiments performed in triplicate, the 1.0 mM (1000 µM; 253.26 µg/mL) penciclovir working solution completely suppressed HSV‐1‐induced cytopathic effect (CPE) formation in Vero cell cultures (Table 8). Representative microscopic images of these findings are presented in Figure 9.

**TABLE 8:** Viral titer values (TCID_50_/mL) of HSV‐1 treated with penciclovir compared with the untreated reference HSV‐1 control.

	1.Trial	2.Trial	3.Trial	A.M	SD	A.M % inhibition
Penciclovir (1 mM)	0	0	0	0	0	100%
HSV‐1 (Vc)	1.78 × 10^8^	1.78 × 10^8^	1 × 10^8^	1.52 × 10^8^	± 4.50×10^7^	—

Abbreviations: A.M., arithmetic mean; SD, standard deviation; VC, virus control.

## 4 Discussion

*M. nigra* is a valuable plant not only for its rich nutritional content and distinctive flavor, but also for its significant role in traditional medicine. It has been widely used as a folk remedy for treating oral and dental disorders, diabetes, hypertension, arthritis, and anemia [7]. In this study, it was found that MNAE has a richer phytochemical content than MNM. The reason why the DPPH and ABTS radical scavenging activities of MNAE (DPPH: 35.53%, ABTS: 70.49%) are higher than those of MNM (DPPH: 17.5%, ABTS: 9.05%) is thought to be due to the higher amounts of polyphenolic compounds. In the previous study, water and methanol extracts of *M. nigra* fruits showed 7% and 21% DPPH radical scavenging activity, respectively, while TPC were found to be 1.6 mcg/ml and 1.4 mcg/ml in terms of gallic acid equivalent [30]. In another study, the TPC and DPPH radical scavenging activity of *M. nigra* fruit was found to be 3.89 ± 0.04 mmol/100 g GAE equivalent and 42.57 ± 3.77 QE equivalent µmol/100 g, respectively [31]. In another study, the DPPH radical scavenging activity of the ethanol extract of black mulberry fruit (IC_50_ value 146.731 µg/mL) was found to be remarkable [32]. In another study, the TPC of *M. nigra* fruits and leaves was measured as 925.33 and 3.07 mg GAE/g extract, respectively. It was determined that the fruit (FRAP: 169.00 ± 4.58 µmoL FeSO_4_.7H_2_O/ g, DPPH: IC_50_ = 13.92 ± 1.62 mg/mL) had a much higher antioxidant value than the leaves. It was determined that the fruits and leaves were rich in chlorogenic acid and protocatechuic acid. It was also determined that the leaves were rich in rutin and the fruits were rich in quercetin [33]. In black mulberry fruit extracts, TPC was found between 90.26 and 118.84 mg GAE/100 g fresh fruit. The content of anthocyanins ranged from 114.83 to 128.68 mg cyanidin‐3‐*O*‐glucoside/100 g fresh fruit. Black mulberry fruit extract showed high antioxidant activity with a DPPH radical scavenging value of 71.41%. Fruit ethanol extract (44.93 ± 2.00%) exhibited good antioxidant activity, as measured by the ABTS assay [34]. In our study, unlike other studies, the DPPH radical scavenging activity was found to be at a moderate level, while the ABTS radical scavenging activity was found to be higher.

Phenolic compounds such as quinic acid, chlorogenic acid, rutin, quercetin, and hesperidin have been detected in black mulberry extract in our LC‐MS/MS study. Other studies have shown that black mulberry exhibits a wide range of biological activities thanks to polyphenolic compounds such as anthocyanins (cyanidin‐3‐glucoside, delphinidin, pelargonidin), flavonoids (quercetin, kaempferol, naringenin), phenolic acids (chlorogenic acid, gallic acid, caffeic acid), and cinnamic acid derivatives (*p*‐coumaric acid, ferulic acid, sinapic acid) [35]. In a phytochemical study on black mulberry molasses, chlorogenic acid (14.1 ± 0.3 mg/100 g dry weight), gallic acid (10.3 ± 0.2 mg/100 g dry weight), syringic acid (0.7 mg/100 g dry weight), neochlorogenic acid (2.3 ± 0.2 mg/100 g dry weight), cyanidin 3‐*O*‐glucoside (in low amounts) were detected [36]. While chlorogenic acid was detected as the major compound in black mulberry fruit in previous studies, quinic acid was found in much higher levels than other phenolic compounds in both molasses (9.936 mg/g extract) and fruit extract (252.086 mg/g extract) in our study. Chlorogenic acid (1.415 mg/g extract) is found to be higher after quinic acid in fruit extract.

Fresh black mulberry fruits were found to have higher total phenol (0.49–57 times higher), flavonoid (0.02–162 fold higher), anthocyanin (6–12209 fold higher) content, and higher antioxidant capacity (0.72–691 fold higher) compared to mulberry products such as mulberry wine, molasses, ice cream, juice, jam, and syrup obtained from the fruit [36]. In another study, a significant decrease in TPC (88%), TFC (89%), TAC (97%), and antioxidant capacity (88%–93%) was observed in jam prepared from black mulberry fruit compared to black mulberry fruit [37]. When compared with other studies, similar results were obtained in our study, indicating that the heat treatment used in preparing molasses from black mulberry fruits can cause significant changes in the content and activity of the fruit. Although anthocyanins are listed in the literature as important bioactive components of plant materials, our study targeted a total of 53 phenolic compounds in LC‐MS/MS analysis, and anthocyanins were not among them. Therefore, factors such as extraction conditions, the chemical composition of the plant material, and the selectivity of the analytical method used may have contributed to the predominant detection of quinic acid in our study.

Chitosan, a polymer known for its mucoadhesive properties, was incorporated into the formulation to ensure localized retention at the site of application. HPMC was used as a film‐forming agent to provide structural integrity to the formulation [38]. As shown in Figure 1, the transparent film represents the blank formulation (F3), the cream‐colored film represents the molasses‐containing formulation (F1), and the pinkish‐red film represents the formulation incorporating black mulberry extract (F2). Characterization of the films revealed that formulations containing molasses had a higher weight per unit, while blank formulations were lighter. The results are meaningful because molasses, extract, and distilled water were added to the formulation on a volumetric basis, and the density of molasses is higher than that of the extract, while the density of the extract is higher than that of distilled water. Furthermore, the weight deviation ranges between 1.01 and 1.40, which is within the appropriate range (Table 6). As seen in Figure 2, the formulations released their content within 5 min. This provides sufficient time for the expected muco‐adhesion from chitosan and indicates that the content can be reached quickly due to the rapid release of the content. When the dispersion rate results were examined, it was seen that they dispersed within sufficient time for mucoadhesive formulations. Although the pH of the formulations are acidic, it is at a level that will not damage the oral and labial structure [27, 28]. When SEM analyses were examined, it was observed that the extract‐containing and blank formulations had similar thicknesses, while the molasses‐containing film formulations were thicker (Figure 3), supporting the weight deviation results. The analysis revealed thin films with smooth surfaces. FTIR analyses showed that the Amin‐I and Amin‐II peaks characteristic of chitosan were also present in the films (Figure 4). However, no differences were observed between the peaks in the FTIR results of the films [27, 28].

In numerous studies, *Morus alba* (white mulberry) has been reported to exhibit antiviral activity against human coronaviruses, picornaviruses, influenza viruses, and herpesviruses (HSV‐1, HSV‐2), which are common causative agents of respiratory and gastroenteric infections [39, 40]. In contrast, research on black mulberry has primarily focused on its organ‐protective properties, as well as its antibacterial, antioxidant, anti‐inflammatory, and anticancer activities, with no prior investigations specifically addressing its antiviral effects [7, 41, 42].

In the present study, the cytotoxic concentrations and antiviral activities of black mulberry were determined in an in vitro system using both fruit extract and molasses. Cytotoxicity and antiviral activity assays demonstrated that the BME exhibited approximately a fivefold higher cytotoxic effect compared to BMM, while also displaying a markedly higher antiviral activity (∼6.67‐fold). This difference is thought to be attributable to the higher content of polyphenolic compounds in the extract.

In an in vitro antiviral study on *Morus alba* leaves, it was reported that *M. alba* extract exhibited 66.7% inhibition of HSV‐1 at an IC_50_ (half‐maximal inhibitory concentration) of 2 µg/mL, while at concentrations of 3.5, 5, and 10 µg/mL, complete (100%) inhibition was observed [16]. Another antiviral study on *M. alba* leaves investigated six stilbene derivatives isolated from mulberry leaves and determined the highest IC_50_ value to be 2.5 µg/mL [43]. In the current study, the antiviral activity of the BME) was measured as ∼97.1% at 0.525 mg/100 µL (5250 µg/mL), while the BMM demonstrated ∼81% inhibition at 0.225 mg/100 µL (2250 µg/mL). The literature indicates that *M. alba* leaf extracts exhibit potent antiviral activity at comparatively lower concentrations. In contrast, our findings highlight that BME and BMM also demonstrate strong antiviral potential. The observed differences in effective concentrations can likely be attributed to variations in plant species, plant organs used, and phytochemical content. Indeed, as with other plants, the phytochemical composition of mulberries can be influenced by species, genetic differences, geographical and environmental factors, and the specific tissues sampled.

Several studies have reported that *M. nigra* contains higher flavonoid concentrations than *M. alba*, which accounts for its stronger antioxidant and anticancer activities [44, 45, 46, 47]. However, the use of *M. alba* leaves in previous antiviral studies, compared with the fruit material used in this study, may explain the relatively higher concentrations required for antiviral efficacy in our experiments. Moreover, the comparatively lower inhibition rates observed in the molasses formulation may be attributed to the loss of phenolic and flavonoid compounds during thermal processing involved in fruit boiling and concentration. In previous studies, caffeoylquinic acids (3,5‐di‐*O*‐CQA, 1‐methoxyoxalyl‐3,5‐di‐*O*‐CQA (1‐MO‐3,5‐di‐*O*‐CQA) and L‐chicoric acid) have been shown to inhibit HIV‐1 integrase activity and replication in vitro [48]. In another study, caffeic acid inhibited HSV‐1 replication in vitro, while quinic acid and its caffeic acid ester, chlorogenic acid, did not [49]. It is not possible to say that the antiviral activity observed in our study is directly due to anthocyanins. Indeed, LC‐MS/MS analysis screened 53 different phenolic compounds, and anthocyanins were not identified among them. However, it is more likely that the different phenolic acids and flavonoids in the extract contribute to both antioxidant and antiviral activity through a synergistic effect. Future studies evaluating the purified compounds individually and in combination will contribute to clarifying the components responsible for the extract's anti‐HSV‐1 activity and any potential synergistic interactions.

The Selectivity Index (SI) is a key parameter used to assess the balance between cytotoxicity and antiviral activity, and it is calculated as the ratio of CC_50_ to IC_50_ [SI = CC_50_ / IC_50_]. In vivo studies of viral infections, a high SI value is considered indicative of a safer antiviral agent [50]. It has been suggested in the literature that for a compound to be considered a promising antiviral candidate for further in vivo investigations, its potential SI value should be ≥10 [51].

In the cytotoxicity assays of this study, the CC_50_ value was determined as 5.83 mg/100 µL for the BMM and 1.26 mg/100 µL for the BME. In antiviral activity assays, concentrations of 0.225 mg/100 µL (BMM) and 0.525 mg/100 µL (BME) achieved inhibitory effects of ∼81% (IC_81_) and ∼97.1% (IC_97_) against HSV‐1, respectively. Based on these data, the theoretically estimated SI values were 25.9 for the BMM and 8.4 for the BME. Considering that actual SI values should be calculated using IC_50_, it is reasonable to assume that BMM and BME may, in fact, possess even higher SI values. Taken together, these findings suggest that the phytochemical components of black mulberry may represent a promising natural antiviral agent for the treatment of cutaneous HSV‐1 lesions.

## 5 Conclusion

In conclusion, it has been demonstrated that *M. nigra* fruit possesses a rich phytochemical content and significant potential in terms of biological activity. Notably, black mulberry extract was found to be superior to black mulberry molasses in terms of TPC, TFC, and TAC content, as determined by LC‐MS/MS analyses. Consequently, its free radical scavenging capacity was also found to be higher. It was observed that significant polyphenolic losses occurred during the processing of products obtained from black mulberry fruit, particularly under heat treatments, resulting in a substantial decrease in biological activity. In addition, because in vitro antiviral activity analyses were evaluated for the first time, BME showed high antiviral activity against Herpes viruses, suggesting that black mulberry may be an important natural source for antiviral treatment research. In the development of functional food and supplement products, the use of fresh black mulberry fruit or extracts obtained by low‐heat treatment may offer higher health benefits. The successful incorporation of BMM and BME into mucoadhesive film formulations provides a preliminary basis for their potential localized application in the orolabial region. However, further comprehensive characterization and in vitro*/in vivo* studies are required to confirm their suitability and performance as topical antiviral delivery systems. These findings support further development and clinical evaluation of mulberry‐based antiviral therapies.

## Author Contributions

**Nuraniye Eruygur**: conceptualization, methodology, investigation, writing – review and editing. **Tuğsen Buyukyildirim**: formal analysis, investigation, methodology, writing‐ original draft. **Sema Arisoy**: data curation, formal analysis, software, visualization, writing – review and editing. **Sercan Palanci**: data curation, formal analysis, software, validation, visualization.

## Conflicts of Interest

The authors declare no conflicts of interest.

## Declaration of Generative AI and AI‐Assisted Technologies in the Writing Process

During the preparation of this revised work, the authors used AI‐assisted writing tools (Gemini / Large Language Models) solely for English language editing, formatting standardization, and refining prose clarity. The authors reviewed and edited the content as necessary and took full responsibility for the content of the publication, including its scientific accuracy, methodology, data interpretation, and original text.

## Supporting information




**Supporting File**: cbdv71863‐sup‐0001‐SuppMat.pdf.

## Data Availability

The data will be available on request to the corresponding author.
