# Supplementary material for: Antiviral Activity of Morus nigra L. Molasses and an Anthocyanin‐Rich Extract Against Herpes Simplex Virus and Development of a Film Formulation
Source: Chem Biodivers. 2026 Nov 9;23(11):e71863. doi: 10.1002/cbdv.71863 (PMC13748926; doi:10.1002/cbdv.71863)
Supplement: Supplementary file 1 [file CBDV-23-e71863-s001.pdf]

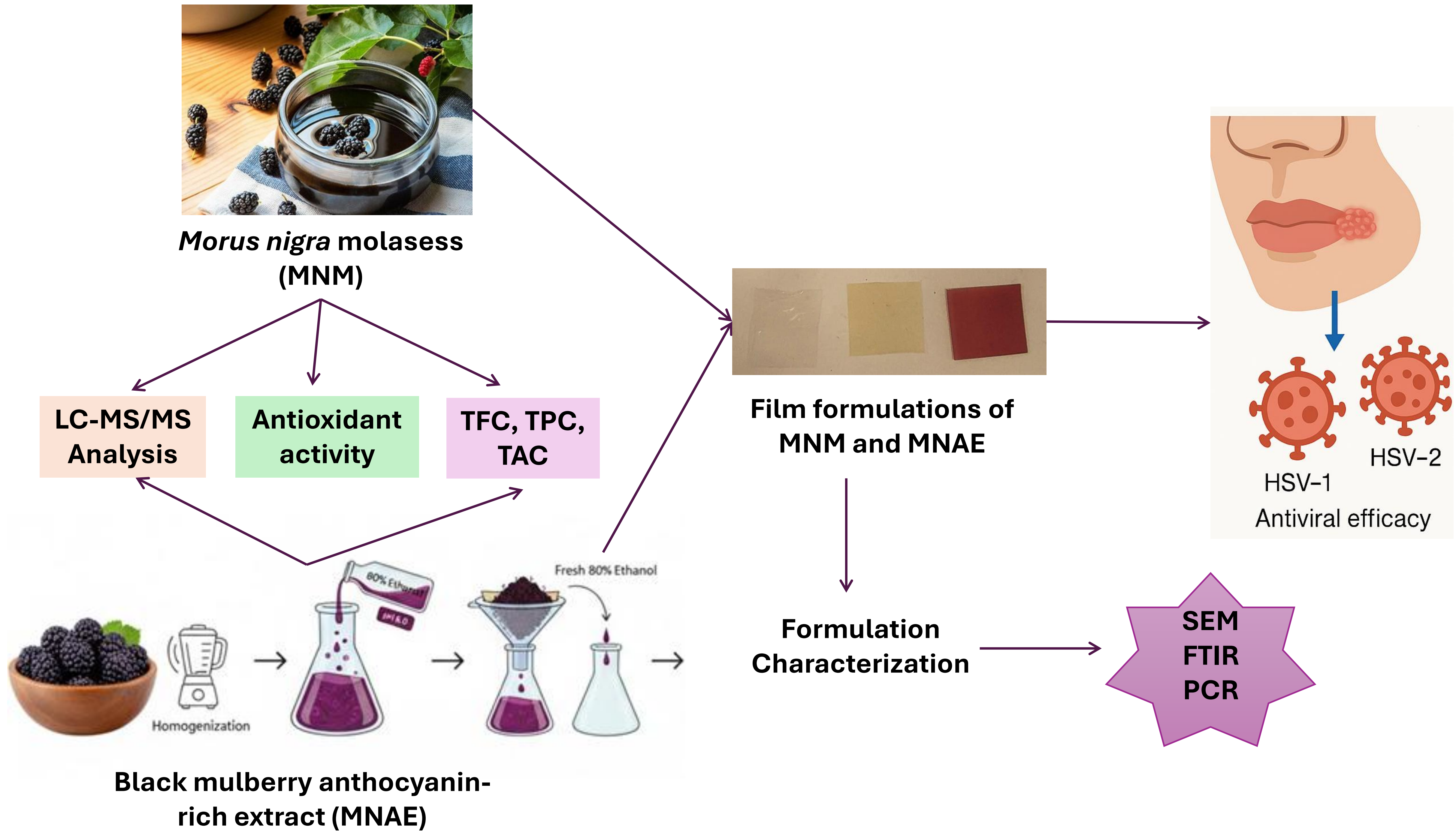

Morus nigra molasess (MNM)
LC-MS/MS Analysis
Antioxidant activity
TFC, TPC, TAC
Fresh 80% Ethanol
80% Ethanol
Homogenization
Black mulberry anthocyanin-rich extract (MNAE)
Film formulations of MNM and MNAE
Formulation Characterization
SEM
FTIR
PCR
HSV-2
HSV-1
Antiviral efficacy
